# Complete Genome Sequences of Staphylococcus epidermidis Myophages Quidividi, Terranova, and Twillingate

**DOI:** 10.1128/MRA.00598-19

**Published:** 2019-06-27

**Authors:** Miranda E. Freeman, Sarah E. Kenny, Amanda Lanier, Katie Cater, Mary C. Wilhite, Paige Gamble, Chandler J. O’Leary, Asma Hatoum-Aslan, Ryland F. Young, Mei Liu

**Affiliations:** a Center for Phage Technology, Texas A&M University, College Station, Texas, USA; b Department of Biological Sciences, The University of Alabama, Tuscaloosa, Alabama, USA; Queens College

## Abstract

Staphylococcus epidermidis is an opportunistic pathogen that commonly colonizes human skin and mucous membranes. We report here the complete genome sequences of three S. epidermidis phages, Quidividi, Terranova, and Twillingate, which are members of the Twort-like group of large myophages infecting Gram-positive hosts.

## ANNOUNCEMENT

Staphylococcus epidermidis is a Gram-positive opportunistic pathogen commonly associated with infections of implanted medical devices, and methicillin-resistant S. epidermidis (MRSE) continues to persist in both the health care and community environments (1, 2). Isolation and characterization of virulent phages active against S. epidermidis may provide an alternative control approach to this bacterium.

Quidividi and Terranova were isolated from a wastewater treatment plant in Tuscaloosa, Alabama, in 2016 with S. epidermidis strain RP62a (2) and strain LM1680, respectively, as the hosts. Twillingate was isolated from a wastewater treatment plant in Northport, Alabama, in 2016 using S. epidermidis strain RP62a (2) as the host. Host bacteria were cultured on tryptic soy broth or agar (Difco) at 37°C with aeration. Phages were cultured and propagated using the soft agar overlay method (3). The three phages were identified as myophages using negative-stain transmission electron microscopy performed at the University of Alabama Optical Analysis Facility as described previously (4). Phage genomic DNA was prepared using a modified Promega Wizard DNA cleanup kit protocol as described previously (4). Pooled indexed DNA libraries were prepared using the Illumina TruSeq Nano low-throughput (LT) kit, and sequencing was obtained from the Illumina MiSeq platform using the MiSeq V2 500-cycle reagent kit following the manufacturer’s instructions, producing 539,431, 484,323, and 538,626 reads for the indexes containing Quidividi, Terranova, and Twillingate, respectively. FastQC 0.11.5 (https://www.bioinformatics.babraham.ac.uk/projects/fastqc/) was used for quality control of the reads. The reads were trimmed with FastX-Toolkit 0.0.14 (http://hannonlab.cshl.edu/fastx_toolkit/download.html) before being assembled to single contigs at 139-, 107-, and 164-fold coverage for Quidividi, Terranova, and Twillingate, respectively, using SPAdes 3.5.0 (5). Contig completion was confirmed with PCR using primers (5′-AGAGGCTATCGCTCTTGAATTAG-3′ and 5′-TGTGTATATTGCTGTCGTGTAGAA-3′ for Quidividi; 5′-GTACAGGTTGGCATCCAGAAT-3′ and 5′-TCCGTGTGCTTGATTACCTTAC-3′ for Terranova; and 5′-TGAATAACTCTGTTAAACGGGCA-3′ and 5′-GGTCGTCACCCTTACGTTTAATT-3′ for Twillingate) facing off the ends of the assembled contigs and Sanger sequencing of the resulting product, followed by manual correction to match the sequencing reads. GLIMMER 3.0 (6) and MetaGeneAnnotator 1.0 (7) were used to predict the protein-coding genes, and tRNA genes were predicted using ARAGORN 2.36 (8). Rho-independent termination sites were identified via TransTerm (http://transterm.cbcb.umd.edu/). Sequence similarity searches were conducted using BLASTp 2.2.28 (9) against the NCBI nonredundant (nr), UniProt Swiss-Prot (10), and TrEMBL databases. InterProScan 5.15-54.0 (11), LipoP (12), and TMHMM 2.0 (13) were used to predict protein function. All analyses were conducted at default settings via the CPT Galaxy (14) and Web Apollo (15) interfaces (https://cpt.tamu.edu/).

Myophages Quidividi, Terranova, and Twillingate possess similar genome sizes (141,446 bp, 141,288 bp, and 142,592 bp, respectively), coding densities (88% to 91%), and low GC contents (each has 28%). The phages share 83% to 88% DNA similarity and are >82% identical to phage phiIPLA-C1C (16), as determined by progressiveMauve 2.4.0 (17). These phages are members of the Twort-like group of large myophages, and they possess multiple self-splicing group I introns in their genomes (18). Introns with precise boundaries were identified in the terminase large subunit, ribonucleotide reductase subunit, double-strand break Mre1 repair protein, endolysin, and DNA polymerase genes. Introns were identified interrupting the tape measure protein-coding regions, but the precise boundaries could not be determined bioinformatically. An intein interrupting the DNA helicase was also identified.

### Data availability.

Quidividi has been deposited in GenBank under accession no. MH321490, SRA no. SRR8788536, and BioSample no. SAMN11260697. Terranova has been deposited in GenBank under accession no. MH542234, SRA no. SRR8788203, and BioSample no. SAMN11259659. Twillingate has been deposited in GenBank under accession no. MH321491, SRA no. SRR8788533, and BioSample no. SAMN11260686. These three phages are located under BioProject no. PRJNA222858.
